# Arginine methylation patterns in LUAD: defining prognostic subtypes and relevance to immunotherapy

**DOI:** 10.1007/s12672-025-02549-5

**Published:** 2025-05-21

**Authors:** Qianyun Shen, Yijie Yang, Maoying Guan, Hegen Li

**Affiliations:** Department of Oncology, Longhua Hospital Affiliated to Shanghai University of Traditional Chinese Medicine, Shanghai, China

**Keywords:** Lung cancer, LUAD, Prognosis, Immune, PRMTs

## Abstract

**Background:**

Lung cancer remains the leading cause of cancer-related death worldwide, with lung adenocarcinoma (LUAD) being the most common subtype. Arginine methylation, driven by protein arginine methyltransferases (PRMTs) has been connected to cancer biology, particularly in modulating cancer immunity. Thus, developing a PRMTs-related prognostic model might help create more personalized treatment plans for LUAD patients.

**Methods:**

We conducted an integrative analysis using multi-omics data from LUAD samples within the TCGA and GEO database, focusing on the expression profiles of nine PRMTs. Employing machine learning, we developed a PRMTs-related prognostic model, to evaluate the clinical and immunological features of LUAD patients.

**Results:**

We stratified 440 LUAD patients into two distinct clusters (PRMTCluster A and B), which exhibited significant differences in prognosis and immune infiltration. The PRMTs-related prognostic model, incorporating genes CLIC6, CLDN2, and BPIFB1, was significantly associated with patient outcomes and immune signature. RT-qPCR showed that the expression level of PRMT1, PRMT3, PRMT4, PRMT5, and PRMT7 was significantly upregulated in H1975 and A549 cells than in BEAS 2B cells.

**Conclusion:**

We developed a PRMTs-related prognostic model for assessing prognosis and immunotherapy responses in LUAD. This model was vital for developing more personalized and effective treatment plans for LUAD patients.

**Supplementary Information:**

The online version contains supplementary material available at 10.1007/s12672-025-02549-5.

## Introduction

Globally, lung cancer is the main cause of cancer-related death. Annually, nearly 2 million individuals are diagnosed with lung cancer, and approximately 1.76 million die from the disease [1]. Lung adenocarcinoma (LUAD) is the most common subtype, comprising about 40% of all lung cancer cases, and immunotherapy is becoming an important emerging therapeutic approach [2, 3]. Immune checkpoint inhibitors (ICIs) have been demonstrated to have the potential to enhance anti-tumor immunity [4]. However, only certain patients are able to derive long-term benefits from immunotherapy, highlighting the necessity for biomarkers to predict treatment outcomes [5]. In addition, LUAD subtypes are also associated with PD-L1 gene expression, Tumor mutational burden (TMB), and potential resistance to immunotherapy [6]. Therefore, it is essential to identify effective biomarkers for immunotherapy for more personalized treatment approaches.

Post-translational modifications (PTMs) of proteins have the potential to enhance protein variety and maintain intracellular balance. However, uncontrolled modifications can potentially result in the development of tumors [7]. Arginine methylation of proteins is a universal PTM facilitated by protein arginine methyltransferases (PRMTs) [8]. Currently, 9 PRMTs have been characterized, which can be divided into three different types based on the enzymatic functions. Type I PRMTs (PRMT1, PRMT2, PRMT3, PRMT4, PRMT6, and PRMT8) are involved in synthesizing asymmetric dimethylarginine. Type II PRMTs (PRMT5 and PRMT9) are responsible for producing symmetric dimethylarginine. Type III PRMT (PRMT7), specifically catalyzes the monomethyl arginine [9, 10]. PRMTs are intricately linked to the modulation of numerous biological processes, encompassing activation and suppression of transcription, cell signaling, cell differentiation, and embryonic development [11]. Notably, PRMTs are involved in the genesis, development, and spread of tumors [12]. PRMTs are recognized as regulators of cancer immunity [13]. Knocking out or inhibiting PRMTs has been shown to enhance the anti-tumor immune response in the treatment of colon cancer, hepatocellular carcinoma, and melanoma [14–16]. Previous research has demonstrated that PRMTs significantly contribute to the progression of lung cancer by influencing essential cellular processes, including gene expression, signaling, and cell cycle [17]. In particular, PRMT5 and PRMT1 play a regulatory role in the proliferation, invasion, chemotherapy resistance, and immune escape of lung cancer cells [18, 19]. However, the research on the role of PRMTs in LUAD, particularly in regulating the tumor immune microenvironment and predicting immunotherapy response, has been relatively limited.

The aim of this research was to address the knowledge gap by investigating the pattern of PRMTs expression in LUAD and its correlation with immunotherapy response. Additionally, we developed a PRMTs-related prognostic model, which can provide personalized prognostic assessment and treatment decisions for LUAD patients.

## Methods

### Data collection

The transcriptome data, clinical information, copy number variations (CNVs) files, and TMB information of LUAD patients were from the Cancer Genome Atlas (TCGA, https://portal.gdc.cancer.gov/repository) database, which served as the training set. This dataset encompasses clinical information from 440 patients. The GSE50081 dataset from the Gene Expression Omnibus (GEO, https://www.ncbi.nlm.nih.gov/geo/) database was defined as the testing set, which contains 127 patients. To ensure data quality and consistency, we excluded samples with incomplete clinical data.

### Consensus clustering analysis of PRMTs

The most suitable cluster quantity and the distribution of LUAD patients based on PRMTs were determined using the “ConsensusClusterPlus” package. The validity of the classification was further affirmed through principal component analysis (PCA) by the “stats” package.

### Prognosis and immune characteristics of different clusters

Differences in survival among different patient subgroups were assessed through Kaplan–Meier (KM) analysis utilizing the “survival” package. The abundance of 28 immune cell subtypes in various subgroups was assessed by the single-sample gene set enrichment analysis (ssGSEA) algorithm. Additionally, we explored changes in the expression of critical immune checkpoint proteins (ICPs) (PD-1, PD-L1, and CTLA-4) in various subgroups.

### Enrichment analysis

We identified the differential expression genes (DEGs) between categorized PRMT clusters utilizing the “limma” package with |log_2_ FC|> 1 and adjusted *P* value < 0.001 [20]. The protein–protein interaction (PPI) network for the DEGs was constructed using the STRING database (https://string-db.org/). By selecting “Homo sapiens” as the species and setting a confidence score threshold of ≥ 0.4, the PPI network was subsequently visualized using Cytoscape (version 3.7.2) [21]. Subsequently, Gene Ontology (GO) and Kyoto Encyclopedia of Genes and Genomes (KEGG) analyses were conducted utilizing the “ggplot2” package to explore various functional roles and pathways significantly associated with the DEGs.

### Establishment and validation of PRMTs-related prognostic model

In this study, the PRMTs-related prognostic model was established. Initially, we performed univariate analysis to identify genes whose expression levels were significantly associated with prognosis, using DEGs and survival data. Then, using the least absolute shrinkage and selection operator (LASSO) method and multivariate analysis, we constructed an optimal prognostic model. Patients were then stratified into low and high PRMTGroups using the median value of the PRMTScore as the cutoff point. KM analysis was employed to assess the difference in survival rates between the two groups. To validate the efficacy of the model, a receiver operating characteristic (ROC) curve was generated by the “timeROC” package. Concurrently, we validated the predictive performance of the PRMTs-related prognostic model using the independent testing set. Furthermore, the prognostic effect of the model on various clinical factors was studied by stratifying LUAD patients based on clinical features.

### Construction of the nomogram

We developed a nomogram to forecast 1-, 3-, and 5-year overall survival (OS) for LUAD patients to validate the accuracy of the PRMT group as an independent prognostic indicator through the “rms” package. Subsequently, calibration curves, ROC curves, and decision curve analysis (DCA) were conducted to ascertain the precision and reliability of the nomogram.

### Immune signature analysis

The abundance of 28 immune cell subtypes was assessed by the ssGSEA algorithm. In addition, we compared differences in the expression levels of ICPs between the two groups stratified by PRMTScore. TMB was divided into low and high TMB groups, categorized by the median TMB value of LUAD patients by the “maftools” package. Furthermore, survival analyses were performed to explore differences between the two groups. To investigate the correlation between immune cells and prognostic genes, Pearson correlation was performed utilizing the “ggplot2” package.

### Cell culture

Normal human bronchial epithelial cells (BEAS-2B) and LUAD cells (A549 and H1975) were procured from the Cell Bank of the Chinese Academy of Sciences and kept in a 5% CO_2_ incubator at 37 °C. The growth medium used was RPMI-1640 (BasalMedia Technologies Co., Ltd., China, L210KJ) containing 10% Fetal Bovine Serum (Lonsera, China, S711-001S).

### RT-qPCR

Total RNA extraction from the cells was performed using an RNA extraction mixture (Servicebio Technology Co., Ltd., China, G3013-100ML). cDNA was synthesized subsequently using a cDNA Synthesis kit (Invitrogen, Thermo Fisher Scientific Inc.) for reverse transcription. The quantification of relative mRNA levels was determined by the 2^−ΔΔCt^ method, with GAPDH mRNA levels serving as the endogenous control for normalization. The primer sequences used are listed in Table 1.

**Table 1:** The PRMTs and PRMTs-associated DEGs sequences

Gene Name	Forward sequence (5ʹ-3ʹ)	Reverse sequence (5ʹ-3ʹ)
PRMT1	CCTGAAAGAGTGTCCCTAGTTGT	TGCAGAAGTAAATGTTCCCATGC
PRMT2	GTCCACTTCCAGAGCCTGCA	CATGAACAGCGTCTGCTTCCA
PRMT3	CAGAACCTGCTCGTCATCTACT	TTCCACACCCAACATCCAAAA
PRMT4	TCGCCACACCCAACGATTT	GTACTGCACGGCAGAAGACT
PRMT5	CGATCAGACCTACTGCTGTCA	CTCGGAGTTCCTGCGAATCT
PRMT6	TACCGCCTGGGTATCCTTCG	CCTGTTCCGGCAACTCTACA
PRMT7	CTCGGAGTTCCTGCGAATCT	CCCGGATACCTTGGTAGTATTT
PRMT8	GGAGCCTCTAGTGGACATCG	CCCATTTTCTTGTGGCACTT
PRMT9	AGCAAGCCAGTGGAACTCTT	TCCTCTCCCATTTCAACCTGG
CLDN2	CCTTTATCACCTCAG CCCGT	GCTACCGCCACTCTGTCTTT
CLIC6	CACGACATCACCCTCTTCGT	AGAGACGCTGAGAAAACGGG
BPIFB1	ATCGGATCCAGCTGATGAAC	AGGAGGCTGGAGTAAGCACA

### Statistical analysis

Statistical analyses and graphical representations were carried out by R v4.1.3 and GraphPad Prism 8.0 software. To determine significant differences between groups, Student’s t-tests were employed. *P* < 0.05 was considered significant.

## Results

### The expression and mutation status of PRMTs in LUAD

**Fig. 1:**
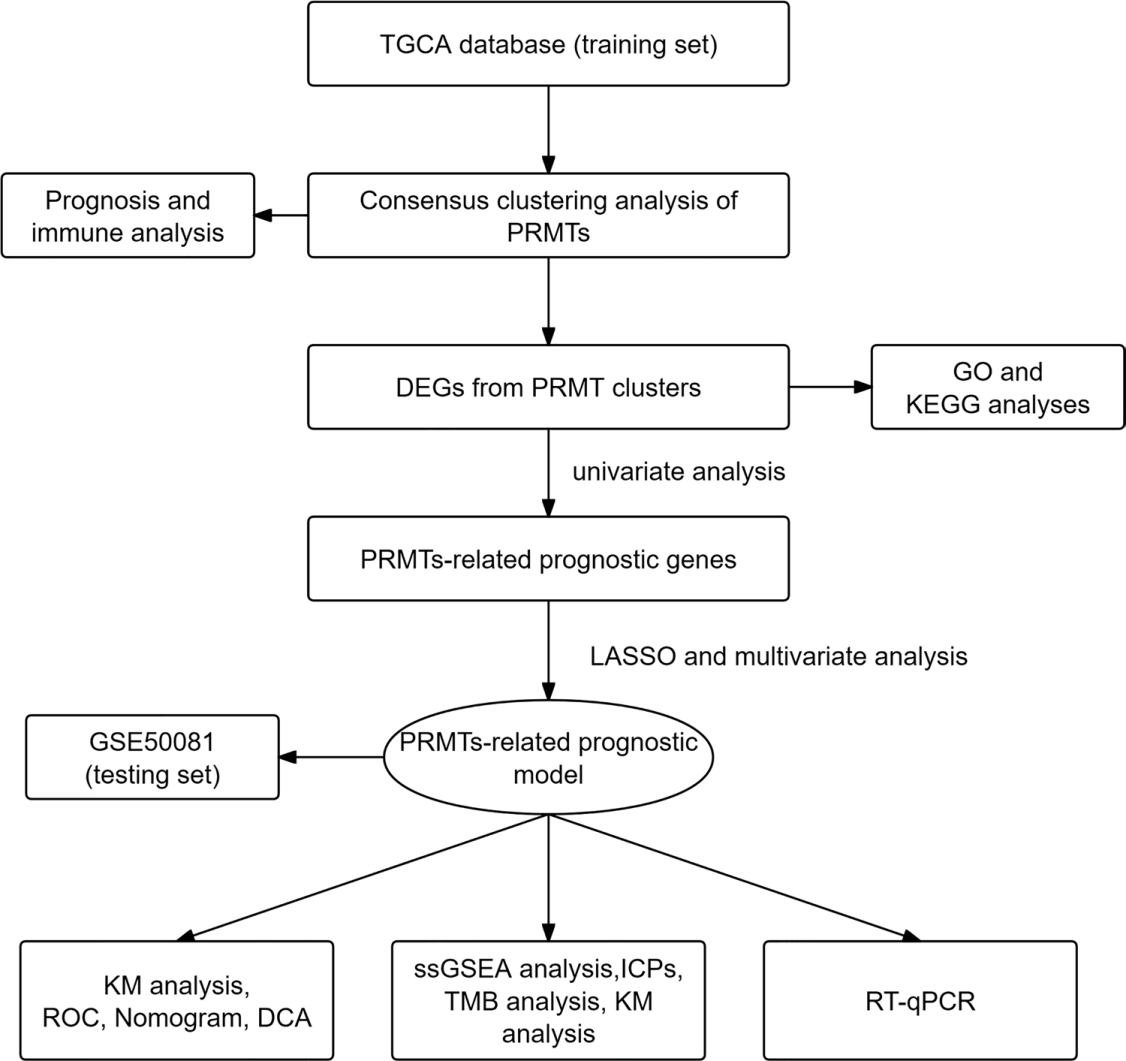
The flowchart of the research design

**Fig. 2:**
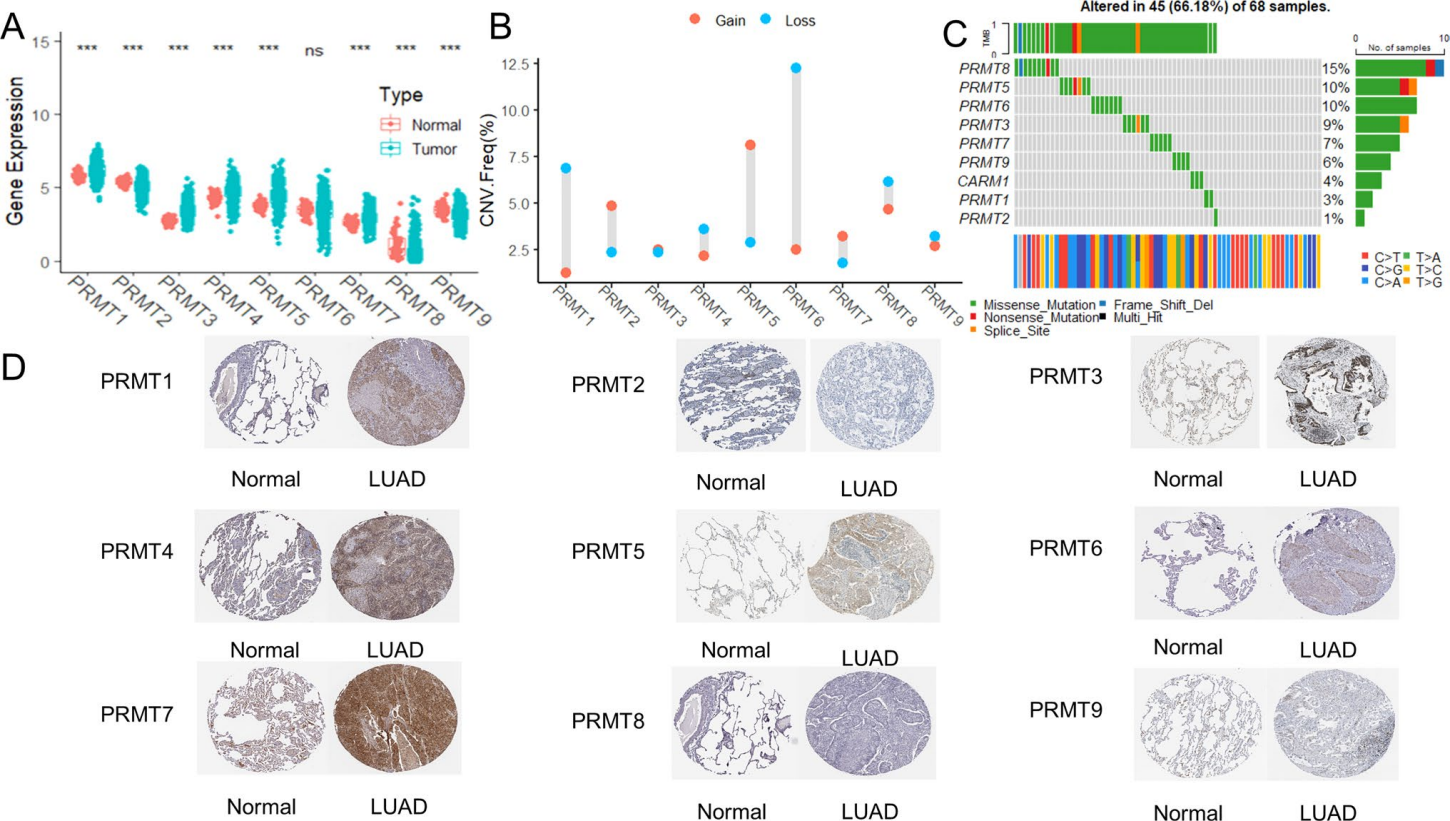
Expression levels of 9 PRMTs in LUAD. **A** The expression levels of 9 PRMTs between LUAD and normal samples. **B** The pattern of CNVs frequency. **C** The somatic mutation frequency. **D** The protein level of 9 PRMTs between LUAD and normal samples from the HPA database

The flowchart of the research design is shown in Fig. 1. To explore the role of PRMTs in LUAD, we analyzed the expression levels of 9 PRMTs (PRMT1, PRMT2, PRMT3, PRMT4, PRMT5, PRMT6, PRMT7, PRMT8, and PRMT9) in tumor and normal lung tissues using TCGA-LUAD data. The expression of PRMT1, PRMT3, PRMT4, PRMT5, and PRMT7 was elevated in tumor samples compared to normal tissue, while PRMT2, PRMT8, and PRMT9 were decreased (Fig. 2A). Subsequently, KM survival analysis was applied to determine the prognostic significance of 9 PRMTs in LUAD patients, and PRMT1, PRMT5, and PRMT8 were identified as associated with patient outcomes (Supplementary Fig. 1). Additionally, we assessed the CNVs for 9 PRMTs within LUAD samples. Notably, amplifications were observed in PRMT2, PRMT3, PRMT5, PRMT7, and PRMT8, while PRMT1, PRMT4, PRMT6, PRMT8, and PRMT9 showed evidence of copy number losses (Fig. 2B). Moreover, we examined somatic mutations occurrence of 9 PRMTs in LUAD specimens, finding that 45 of 68 cases (66.18%) had genetic alterations (Fig. 2C). Notably, PRMT1, PRMT3, PRMT4, PRMT5, and PRMT7 were more highly expressed in LUAD tissues than in normal lung tissues from the Human Protein Atlas (HPA) database (Fig. 2D). Collectively, these results highlight the frequent genetic alterations and somatic mutations involving PRMTs in LUAD, suggesting its potential role in disease pathology.

### Differential subgroups identification of PRMTs

**Fig. 3:**
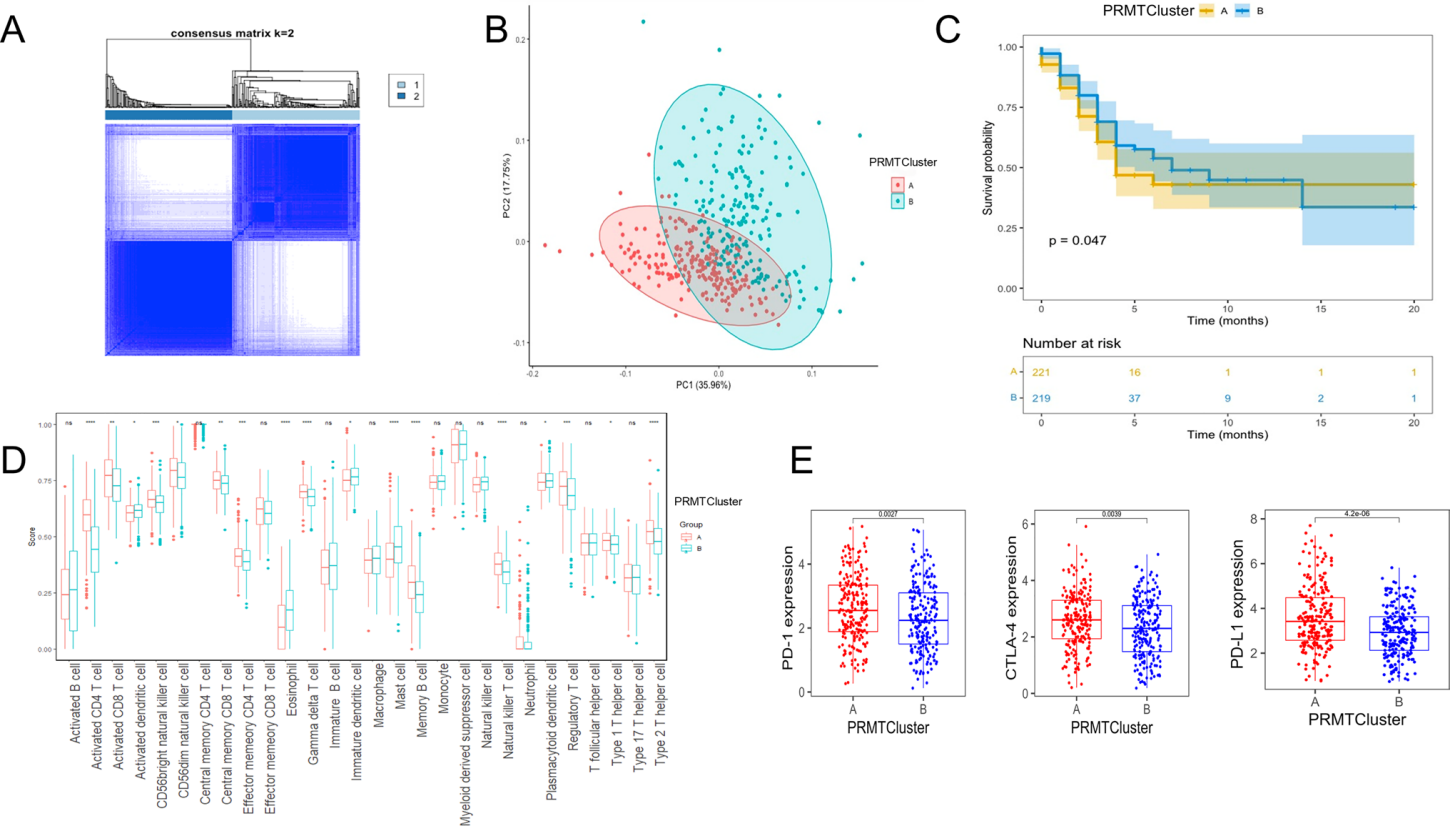
The classification of two PRMTClusters. **A** A consensus matrix heatmap for delineating the two PRMTClusters. **B** PCA for the two subgroups. **C** KM curves of OS for patients in PRMTClusterA and PRMTClusterB. **D** Abundance of 28 immune cells in PRMTClusters. **E** Expressions of PD-1, PD-L1, and CTLA-4 in two PRMTClusters

We performed a consensus clustering analysis to classify LUAD patients into distinct patterns of arginine methylation modifications, referred to as PRMTClusters. The optimal number of clusters was determined to be 2 based on the cumulative distribution function (CDF) curve trend and the inflection point of the delta area curve (Supplementary Fig. 2). Subsequently, patients were allocated into PRMTCluster A (221 patients) and PRMTCluster B (219 patients) (Fig. 3A; Supplementary Table S1). Utilizing the expression profiles of 9 PRMTs, PCA validated significant differences in subgroup distribution in each group (Fig. 3B). Moreover, significant differences were observed between the survival curves of the two groups (*P* < 0.05), indicating that PRMTCluster B had superior survival outcomes at multiple time points. However, the presence of intersecting survival curves suggests that the survival rates of the two groups may fluctuate over time. Specifically, during certain periods, the survival rate of PRMTCluster A may surpass that of PRMTCluster A (Fig. 3C). We then utilized the ssGSEA algorithm to compare the infiltration levels of 28 immune cell populations between the two PRMTclusters. Our analysis revealed substantial differences in immune cell enrichment across various cell types (Fig. 3D). Specifically, PRMTCluster A had higher levels of regulatory T cells (Tregs) infiltration. Notably, PRMTCluster A showed elevated expression levels of PD-1, PD-L1, and CTLA-4 (Fig. 3E). In summary, by dividing LUAD patients into two clusters based on the expression patterns of 9 PRMTs, we were able to identify significant differences in prognostic outcomes and immune cell profiles within two PRMTClusters.

### Enrichment analysis of DEGs related to PRMTClusters

**Fig. 4:**
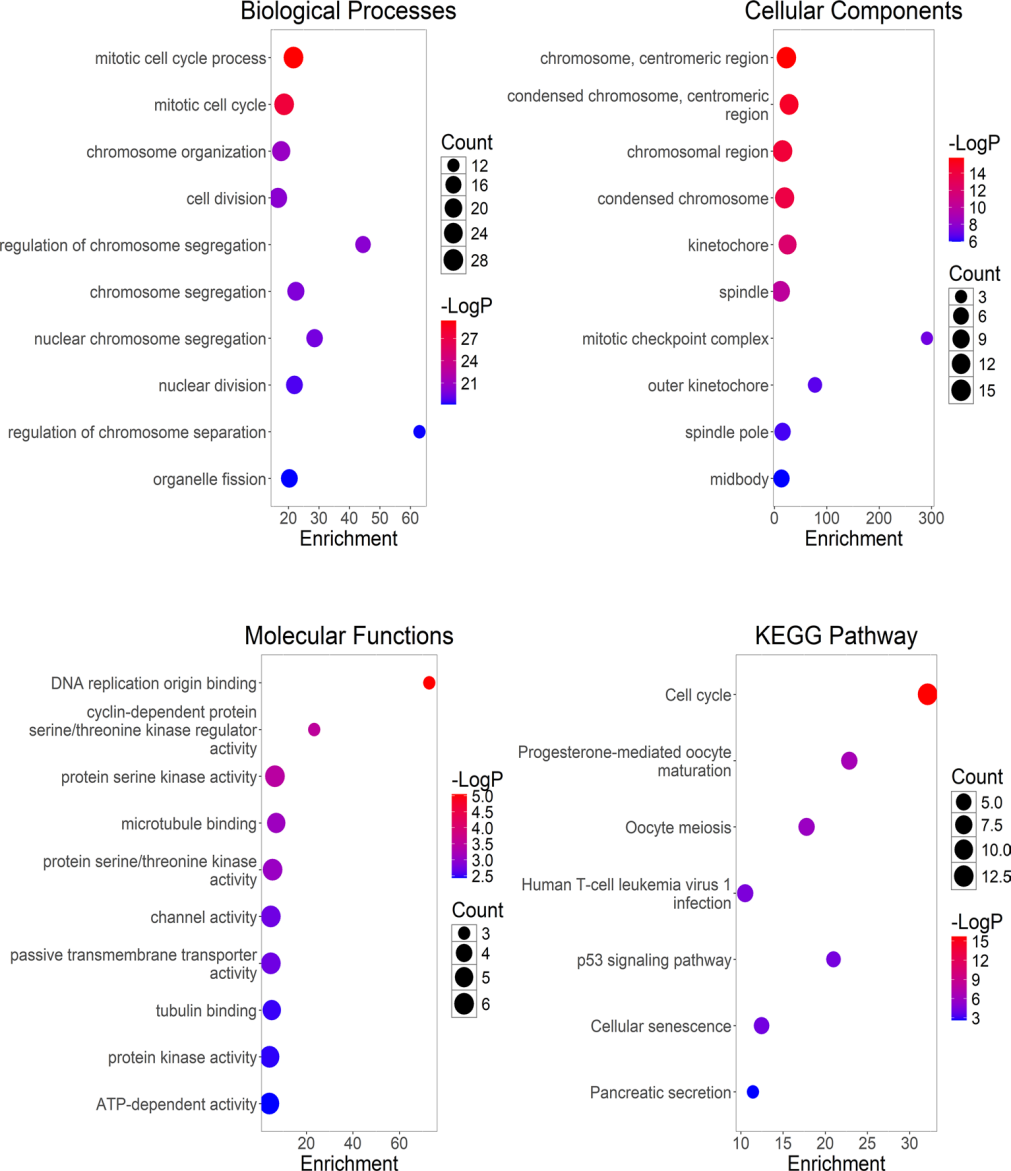
The enrichment analysis of 79 DEGs

To understand the biological differences among LUAD samples classified by PRMT expression patterns, we pinpointed 79 DEGs related to PRMTClusters utilizing the “limma” package (Supplementary Table S2). The PPI network for 79 DEGs was constructed by the STRING database (Supplementary Fig. 3). Subsequently, we performed GO analyses, and the results revealed that the enriched GO categories mainly included mitotic cell cycle process, chromosome, centromeric region, DNA replication origin blinding, etc. Furthermore, the KEGG categories are associated with the cell cycle and P53 signaling pathway, highlighting the crucial pathway of PRMTs in the occurrence and development of LUAD (Fig. 4).

### Construction of a PRMTs-related prognostic model

**Fig. 5:**
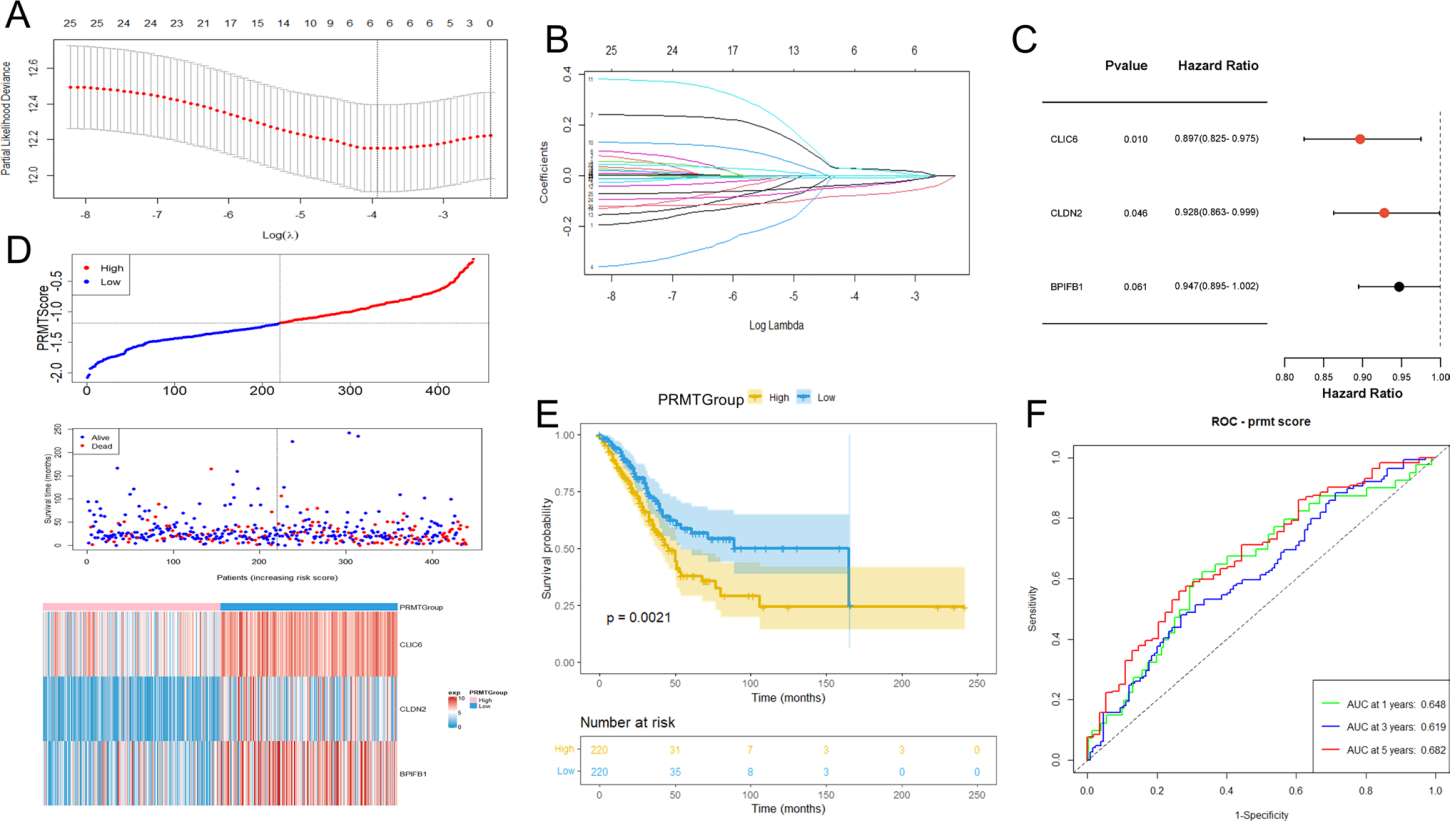
Construction of a PRMTs-related prognostic model. **A**, **B** LASSO analysis. **C** Three genes were identified by multivariate analysis. **D** The distributions, survival status, and expressions of the three prognostic genes in the training set. **E** KM analysis for two PRMTGroups patients in the training set. **F** ROC curves analysis in the training set

To further assess the association of DEGs with prognosis, we employed univariate analysis. This approach identified 25 genes whose expression was significantly correlated with patient survival outcomes (*P* < 0.05) (Supplementary Table S3). We then designed a PRMTs-related prognostic model aimed at prognostic prediction in patients with LUAD, which was constructed based on 25 prognosis-associated DEGs. Utilizing LASSO and multivariate analyses, we established an optimal predictive model (Fig. 5A–C). Within this model, three genes, namely CLIC6, CLDN2, and BPIFB1, were identified as having the most significant prognostic influence on the development of a predictive tool. Notably, elevated expression levels of CLIC6, CLDN2, and BPIFB1 were associated with a poorer prognosis. There was a significant correlation between increased PRMTScore and increased mortality rates in the training set (Fig. 5D). In Fig. 4E, the KM survival curves for the high-risk and low-risk groups are presented. The difference of survival curves between the two groups in the training set was statistically significant (*P* < 0.05), indicating that patients in the low-risk group showed a better survival outcome in a longer period of time. In the training set, the AUC for 1-, 3-, and 5-year OS were 0.648, 0.619, and 0.682, respectively (Fig. 5F). The verification results of the GSE50081 cohort are basically consistent with those of the TCGA-COAD cohort. There were significant survival differences between the two groups (*P* < 0.05) in the testing set (Supplementary Fig. 4B). In the testing set, the AUC for 1-, 3-, and 5-year OS were 0.518, 0.629, and 0.63, respectively (Supplementary Fig. 4C). Interestingly, patients in PRMTCluster A showed a higher PRMTScore (Supplementary Fig. 4D).

### Correlation between clinical features and the prognosis

**Fig. 6:**
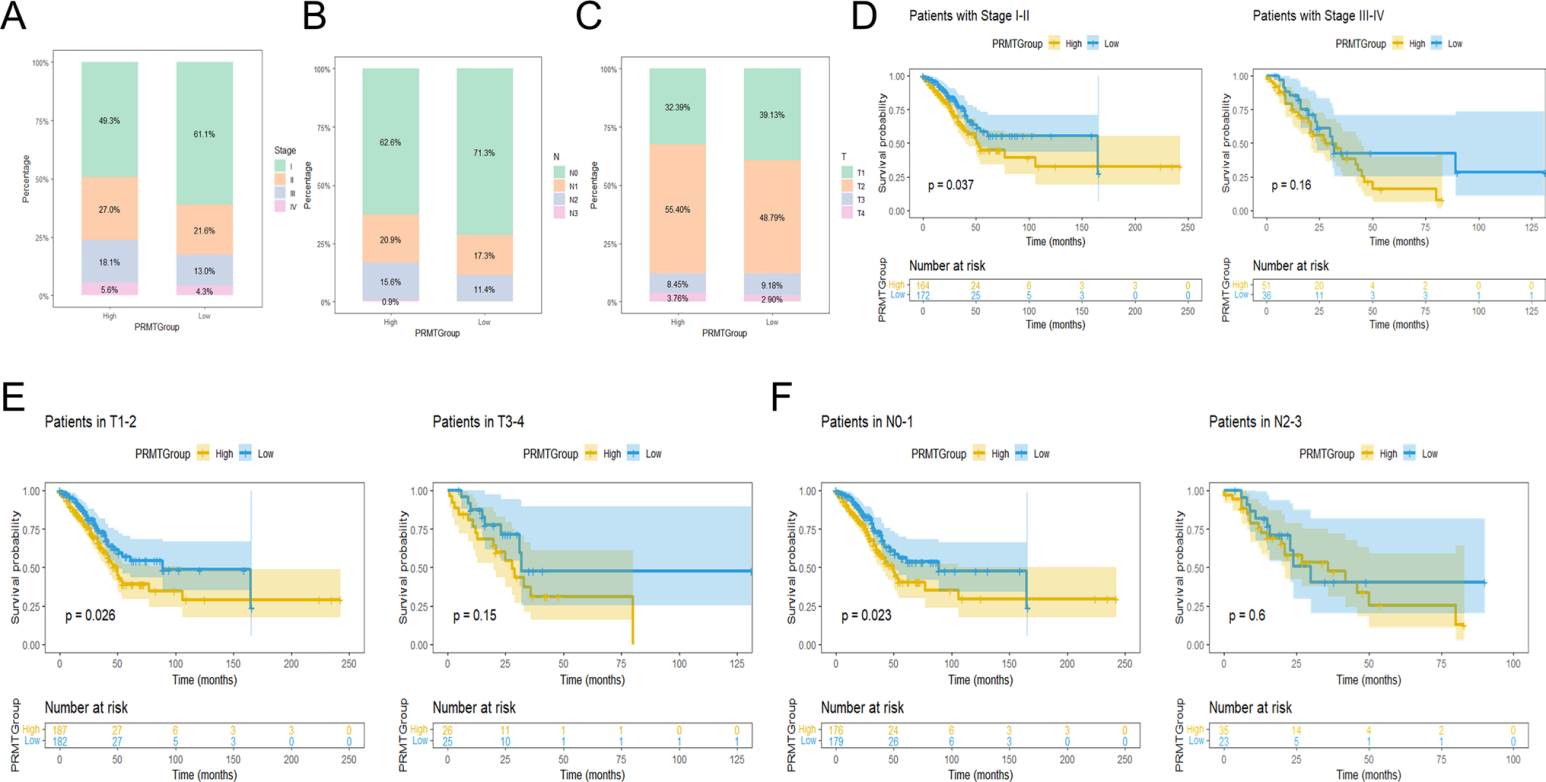
Correlation between clinical features and prognosis. **A**–**C** The proportion of clinical features (grade, T, N, and M) of two PRMTGroups. **D**–**F** KM analysis of two PRMTGroups patients with clinical factors (stage, T, and N)

To assess the accuracy of the prognostic model, KM analyses were applied to different subgroups of clinical features. The low PRMTScore patients were more likely to have lower stage, T, N, and M (Fig. 6A–C). Importantly, high PRMTScore patients tended to have poorer clinical outcomes, especially in the early stages of lung cancer (T1 and T2, N0 and N1, M0, stage I and stage II) (*P* < 0.05) (Fig. 6D–F; Supplementary Fig. 5C–E). Collectively, these findings highlight the accuracy and consistency of our model in predicting prognostic outcomes for LUAD patients across different clinical features.

### Establishment of the nomogram

**Fig. 7:**
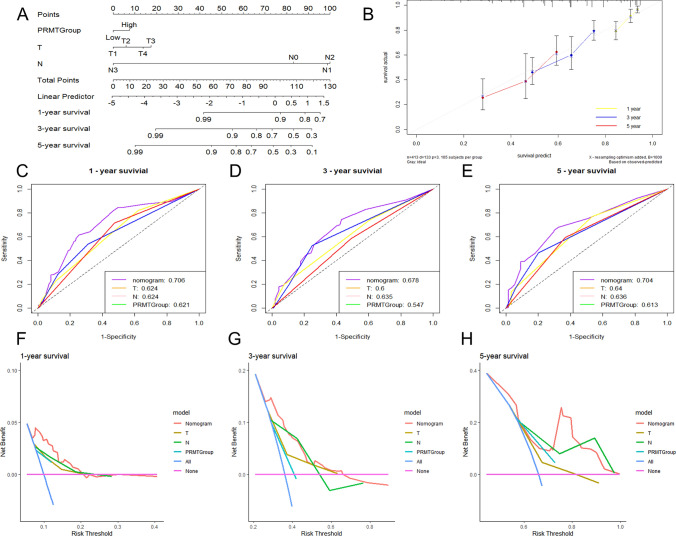
Construction of a nomogram. **A** The nomogram curves. **B** The calibration curves. **C**–**E** The ROC curves. **F**–**H** The DCA curves

The univariate and multivariate analysis of clinical features and PRMTScore demonstrated that T, N, and PRMTScore were the independent prognostic factors for LUAD patients (Supplementary Fig. S6). We then developed a nomogram designed to estimate the 1-, 3-, and 5-year OS rates for LUAD patients (Fig. 7A). This nomogram's predictive accuracy was confirmed by a calibration plot (Fig. 7B). The AUC values of the nomogram for 1-, 3-, and 5-year survival rate were 0.706, 0.678, and 0.704, respectively, demonstrating that the model had good prediction ability (Fig. 7C–E). Additionally, DCA confirmed the nomogram model had practical clinical utility (Fig. 7F–H). These findings collectively suggest that our nomogram serves as a robust tool for prognostic prediction in LUAD and could be integrated into personalized patient management plans to improve treatment strategies.

### Immune signature of the model

**Fig. 8:**
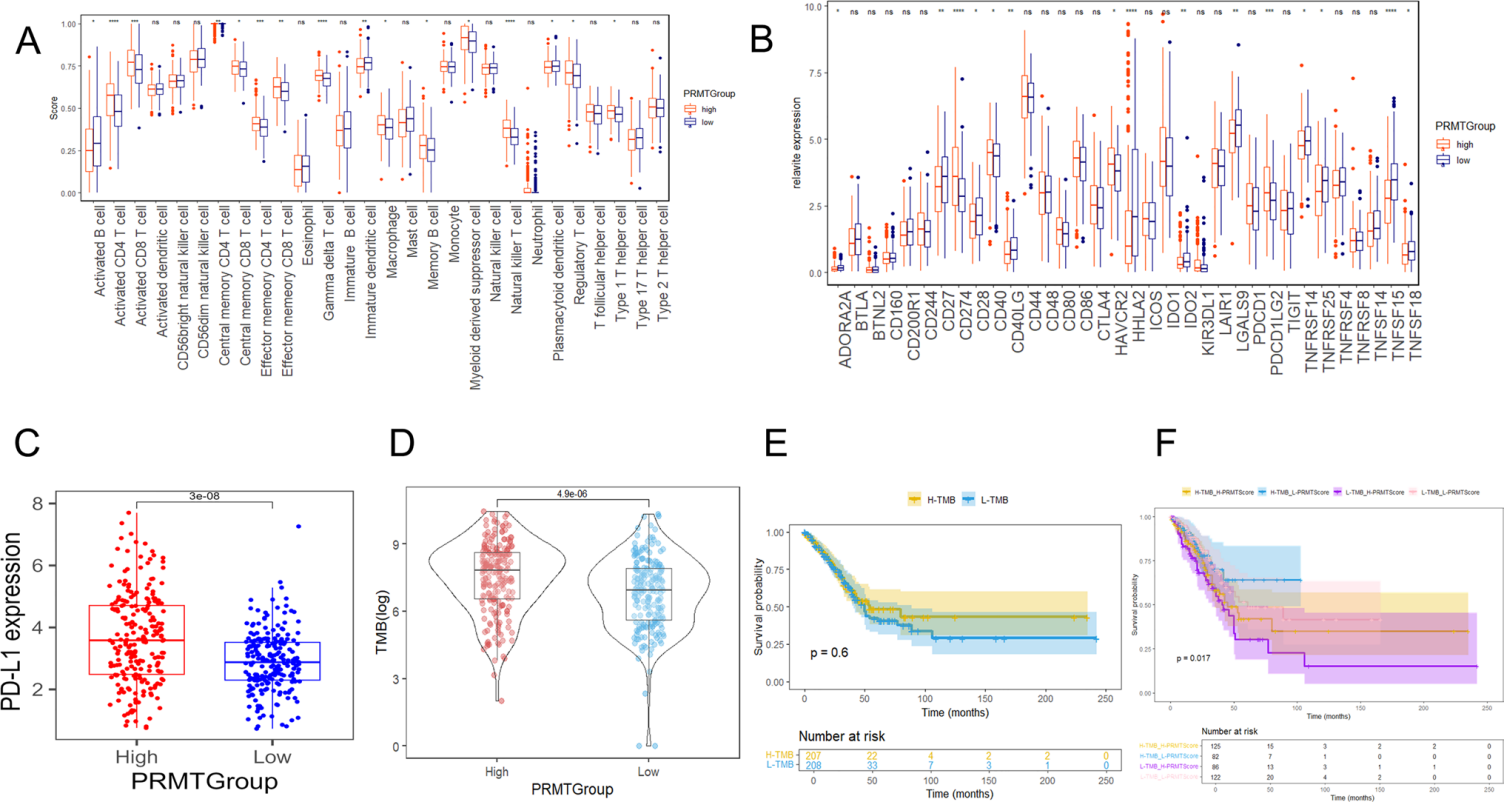
Evaluation of the immune signature of the prognostic model. **A** The bundance of 28 immune cells in two PRMTGroups. **B** Expressions of ICPs in two PRMTGroups. **C** Expressions of PD-L1 in two PRMTGroups. **D** Expressions of TMB in two PRMTGroups. **E** KM analysis of LUAD patients in the high- and low-TMB groups. **F** KM analysis of LUAD patients in the two risk groups combining two TMB groups

Considering the effect of the model on immunotherapy, we conducted immune cell infiltration among two PRMTGroups. The high PRMTScore group showed increased infiltration of multiple immune cells, including activated CD4^+^ T cells, activated CD8^+^ T cells, and Tregs, etc. (Fig. 8A). Additionally, the expression of ICPs across different PRMTGroups was compared. The results revealed that there were significant differences in the expression of several ICPs (Fig. 8B). Specifically, the expression levels of PD-L1 were elevated in the high PRMTScore group (Fig. 8C). We found that the TMB values of the high PRMTScore group were higher than those of the low PRMTScore group, suggesting that PRMTScore may be related to TMB (*P* < 0.05) (Fig. 8D). To more fully evaluate the predictive effect of PRMTScore on immunotherapy response, we further combined TMB and PRMTScore for a comprehensive analysis. There was no significant difference in TMB between the two groups (*P* > 0.05) (Fig. 8E). High TMB group combined with low PRMTScore had the highest OS rate (*P* < 0.05) (Fig. 8F). Moreover, correlation analysis indicated that PRMTs-related genes (CLIC6, CLDN2, and BPIFB1) were associated with immune cells (Supplementary Fig. S7).

### Identification of prognostic PRMTs expression

**Fig. 9:**
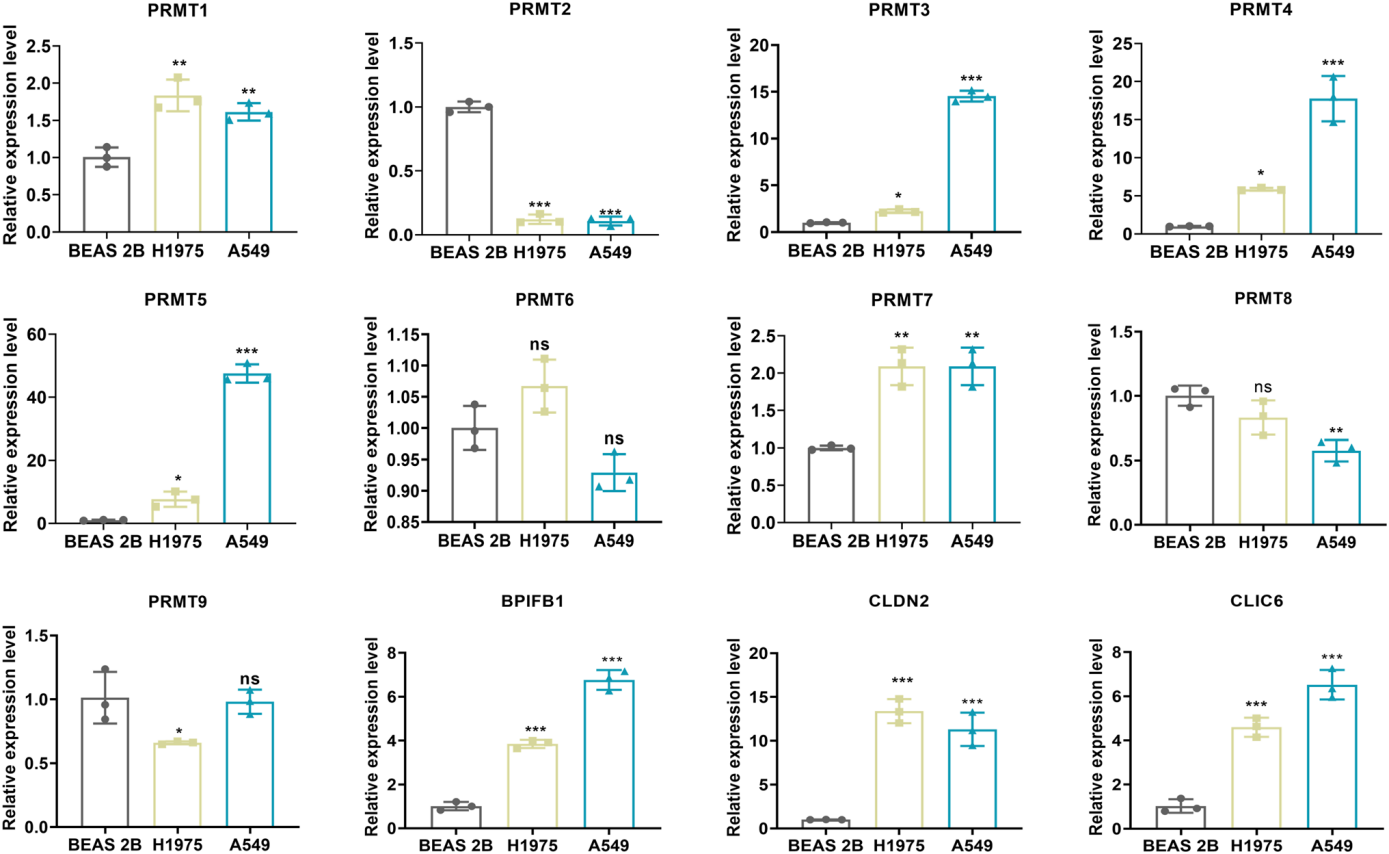
Analysis of mRNA levels of 9 PRMTs and three PRMTs-associated DEGs (CLIC6, CLDN2, and BPIFB1) in LUAD cell lines (H1975 and A549) and normal BEAS 2B cells by RT-qPCR. * *P* < 0.05, ** *P* < 0.01, *** *P* < 0.001

We performed RT-qPCR and found that the expression level of PRMT1, PRMT3, PRMT4, PRMT5, and PRMT7 was significantly upregulated in H1975 and A549 cells than in BEAS 2B cells (*P* < 0.05), which were consistent with our predictions. Conversely, PRMT2 was downregulated in H1975 and A549 cells (*P* < 0.001). PRMT8 was downregulated only in A549 cells, and PRMT9 was downregulated only in H1975 cells (*P* < 0.05). Moreover, the expression of three PRMTs-associated DEGs (CLIC6, CLDN2, and BPIFB1) was significantly upregulated in H1975 and A549 cells than in BEAS 2B cells (*P* < 0.001) (Fig. 9).

## Discussion

LUAD is the predominant histological subtype of lung cancer and causes the highest cancer-related mortality worldwide [22]. Immunotherapy for lung cancer significantly improves prognosis [23]. Specifically, the introduction of ICIs has significantly extended survival for lung cancer patients [24]. However, immunotherapy is limited by the heterogeneity of the tumor genome and differences in the immune microenvironment [25], resulting in wide variation in treatment between individual patients [26]. Hence, identifying biomarkers is an important area for improving immunotherapy efficacy.

As regulators of the epigenome, PRMTs are pivotal in governing a range of cellular processes, including the maintenance of pluripotency, cellular differentiation, proliferation, survival, and apoptosis [27]. Interestingly, various studies have documented that PRMTs are capable of modulating the process of tumorigenesis in lung cancer. For example, PRMT6 inhibits LUAD development by activating p18 expression in vivo [28]. Previous studies have indicated that PRMT1 and PRMT5 serve as signature markers that are significantly related to prognostic outcomes and immune profiles in patients with LUAD [29]. Additionally, we assessed the expression levels of 9 PRMTs across various LUAD cell lines, and we found that PRMTs exhibit distinct differential expression patterns in LUAD. These studies offer compelling evidence that PRMTs have the potential as promising biomarkers for prognosis and as therapeutic targets in lung cancer. However, few other PRMT members have been studied in the field of lung cancer research.

In our research, we utilized the consensus clustering algorithm to divide LUAD patients into two subgroups characterized by arginine methylation modification, called PRMTCluster A and B. These subgroups showed significant differences in prognostic outcomes and immune cell profiles. Notably, PD-1, PD-L1, and CTLA-4 were significantly overexpressed in PRMTCluster A, suggesting immune escape may occur in this subgroup. PD-1 and PD-L1 are pivotal immune checkpoint molecules that modulate the immune response by inhibiting T cell activation and function. In the tumor microenvironment, tumor cells highly express PD-L1, which transmits inhibitory signals by binding to the PD-1 receptor on the surface of T cells, leading to T cell exhaustion, thus achieving immune escape [30]. CTLA-4 can bind CD80/CD86 and inhibit T cell activation and proliferation [31]. Given these findings, patients in PRMTCluster A may be more responsive to immunotherapy and may benefit from prioritization for PD-1/PD-L1 inhibitors combined with CTLA-4 inhibitors. Conversely, patients in PRMTCluster B may need other strategies, such as combination chemotherapy or other targeted therapies. Collectively, these results indicate that PRMTs may influence the progression of LUAD by modulating immune cell infiltration and immune escape in the tumor microenvironment. PRMTs may be key targets for the potential treatment of LUAD.

To assess the role of PRMTs in individual LUAD patients, we constructed a PRMTs-related risk model. Utilizing univariate analysis, we further refined our findings to isolate 25 genes associated with prognosis. Utilizing LASSO and multivariate analysis, we then constructed a PRMTs-related prognostic model containing three PRMTs-related DEGs (CLIC6, CLDN2, and BPIFB1 and calculated the PRMTScore. To ascertain the predictive accuracy of the model, we generated ROC curves. The AUC values (0.648, 0.619, and 0.682) for the 1-, 3-, and 5-year OS, respectively, suggested that our prognostic model had moderate accuracy in predicting survival outcomes. Future studies could further optimize the model to enhance its accuracy and reliability. Patients in the high PRMTScore group tended to have poorer clinical outcomes, especially during the early stage, T, N, and M. This finding suggests that the high PRMTScore may be associated with tumor progression and poor prognosis and implies that Patients in the high PRMTScore group may require early monitoring, early intervention, and other therapeutic measures. Finally, we constructed a nomogram to integrate the PRMTScore with independent prognostic clinical features (stage, T, and N), which can predict individual patients’ prognosis and formulate more precise treatment plans.

The PRMTs-related prognostic genes (CLIC6, CLDN2, and BPIFB1) have been proven to participate in the regulation of the immune response. Previous research has indicated that the silencing of the CLDN2 gene results in the production of interleukin-17, a decrease in T-cell activation, and a limitation of intestinal damage [32]. BPIFB1 has been shown to promote the spread of breast cancer that tests positive for hormone receptors, the process that involves driving macrophage M2-like polarization [33]. The overexpression of BPIFB1 markedly suppressed the expression of PD-L1, which in turn inhibited apoptosis and resulted in an increase in the expression of CD8 T cells in nasopharyngeal carcinoma cells [34]. In our study, the expression of CLIC6, CLDN2, and BPIFB1 was significantly upregulated in H1975 and A549 cells, suggesting that high expression of these genes may influence the progression of lung cancer through immune regulation. PRMTs may promote tumor development by influencing immune escape through the CLIC6/CLDN2/BPIFB1 axis. However, the specific mechanisms by which PRMTs regulate these target genes to modulate immune responses are currently lacking in literature support and require further exploration in future research.

Next, we investigated the immune characteristics of the high and low-PRMTScore groups in the prognostic model. The infiltration level of immune cells and the expression of PD-L1 were higher in the high PRMTScore group. We found that the TMB values of the high PRMTScore group were higher than those of the low PRMTScore group, suggesting that PRMTScore may be related to TMB, and the high PRMTScore may reflect the high mutation load of the tumor cells, resulting in more immune cell infiltration. Studies have shown that high TMB usually produces more neoantigens, enhancing immune system recognition and anti-tumor response, and the higher the TMB level, the more likely immunotherapy benefits [35, 36]. However, the combination of high TMB and low PRMTScore was associated with higher OS rates, which seems paradoxical. We speculate that the high PRMTScore group may lead to recruitment and activation of immune cells through epigenetic modifications such as arginine methylation. The high PRMTScore group may promote tumor cells to secreta chemokines (such as CXCL9/10), recruit immune cells such as CD8+ T cells, and induce immune escape of PD-L1 expression [37]. In addition, high expression of PD-L1 induces T cell failure through the PD-1 pathway and may not translate into a survival advantage [38]. The low PRMTScore group has weaker immunosuppressive signals in the tumor microenvironment, allowing the immune system to more effectively recognize and eliminate tumor cells, creating a synergistic effect with high TMB antigenicity. In summary, PRMTScore may be only one factor in the tumor immune microenvironment, and it may interact with a variety of other factors that collectively influence tumor immune response and prognosis. When PRMTScore is low, the immune activation potential of high TMB can be fully released and translated into survival benefits. This finding suggests that PRMTScore can be used as a marker to accurately screen people for benefit from immunotherapy. Therefore, we recommend stratifying patients with PRMTScore for immunotherapy. Despite the increased infiltration of immune cells in the high PRMTScore group, the suppression of the immune microenvironment may lead to the limited efficacy of PD-1 inhibitors, so combination therapy is more effective, such as PD-L1 inhibitors combined with CTLA-4 inhibitors, which can reverse the Treg-mediated immunosuppression by blocking CTLA-4 [39]. In combination with PRMTs inhibitors (such as GSK3326595), PD-L1 can be down-regulated, reducing the proportion of Tregs [40]. Patients in the PRMTScore group had lower levels of immune cell infiltration, and the combination of high TMB and low PRMTScore was associated with higher OS rates, suggesting that patients in the low-risk group may benefit more from immunotherapy and be the best candidates for monotherapy with anti-PD-1 /PD-L1 drugs. However, if the tumors in the low-risk group progress slowly and are less aggressive, standard chemotherapy may be a suitable option because it can effectively control tumor progression but may have more predictable side effects than immunotherapy. Concurrently, the PRMTs prognostic model is combined with the existing clinical staging system (such as AJCC staging) to detect the change of PRMTScore through ctDNA for precise treatment.

Compared with the existing LUAD prognostic model, our PRMTScore prognostic model is based on the methylation modification pattern of PRMTs, which has been less explored in previous studies on LUAD. Secondly, our model integrates the interactions among multiple PRMTs using machine learning algorithms to enhance the accuracy of prognosis prediction. Additionally, the PRMTScore model incorporates an assessment of immune characteristics and predicted therapeutic response to immunotherapy. Analyzing differences in immune infiltration and expression of ICPs across different clusters of PRMTs can provide a more comprehensive reference for the personalized selection of immunotherapy for LUAD.

However, our study has some limitations. Firstly, this research is analyzed based on the open database. Notably, the sample size may be insufficient, potentially introducing bias to our findings. To address this limitation, future studies will employ a prospective design to collect more clinical samples from lung cancer patients. Additionally, the predictive ability of this model for immune treatment responses needs to be prospectively validated. We will also take into account other factors that may affect immune treatment responses, such as TMB, and control for them in future research. Secondly, the specific regulatory mechanisms of PRMTs on CLIC6/CLDN2/BPIFB1 have not been elucidated and verified. We plan to conduct in vitro cell culture experiments and in vivo animal models to investigate the specific biological functions of PRMTs and consider using the CRISPR/Cas9 technology to further clarify the direct interactions between PRMTs and target genes. With these complementary studies, we hope to overcome the limitations of the current research and provide a stronger foundation for future clinical applications.

## Supplementary Information

Supplementary Material 1: Fig. S1. The prognostic analysis of PRMTS in LUAD patients by the GEPIA2 database.

Supplementary Material 2: Fig. S2. Identification of arginine methylation modification patterns based on the expression levels of the 9 PRMTs. (A-E) Consensus matrices of LUAD patients from k = 2 to k = 6. (F-H) The CDF curves plot, delta plot, and tracking plot corresponding to the consensus matrices from k = 2 to k = 6.

Supplementary Material 3: Fig. S3. The PPI network of 79 DEG.

Supplementary Material 4: Fig. S4. Verification of PRMTs-related prognostic model in the testing set. (A–C) The distributions, survival status, and expressions of the three prognostic genes in the testing set. (B) KM analysis for two PRMTGroups patients in the testing set. (C) ROC curves analysis in the in the testing set. (D) Differences of PRMTScore among two PRMTClusters.

Supplementary Material 5: Fig. S5. Correlation between clinical features and the prognosis. (A-B) The proportions of clinical features (age and gender) of two PRMTGroups patients. (C-E) KM analyses for two PRMTGroups patients with clinical features (age, M, and gender).

Supplementary Material 6: Fig. S6. Univariate and multivariate Cox regression analyses based on the PRMT group and other clinical features in LUAD. (A) The univariate analysis. (B) The multivariate analysis.

Supplementary Material 7: Fig. S7. Correlation analysis of the PRMT-related genes (CLIC6, CLDN2, and BPIFB1) and immune cells.

Supplementary Material 8: Table S1. The distributions of LUAD patients in PRMTClusters and PRMTGroups.

Supplementary Material 9: Table S2. The DEGs between the PRMTClusters.

Supplementary Material 10: Table S3. The univariate analysis of 25 DEGs.

## Data Availability

This study was utilized from the TCGA database and the GEO database. The necessary data for analysis can be found in the manuscript or the Supplementary Information file.
