# Supplementary figures and images for: Arginine methylation patterns in LUAD: defining prognostic subtypes and relevance to immunotherapy
Source: Discov Oncol. 2025 May 21;16:853. doi: 10.1007/s12672-025-02549-5 (PMC12095734; doi:10.1007/s12672-025-02549-5)

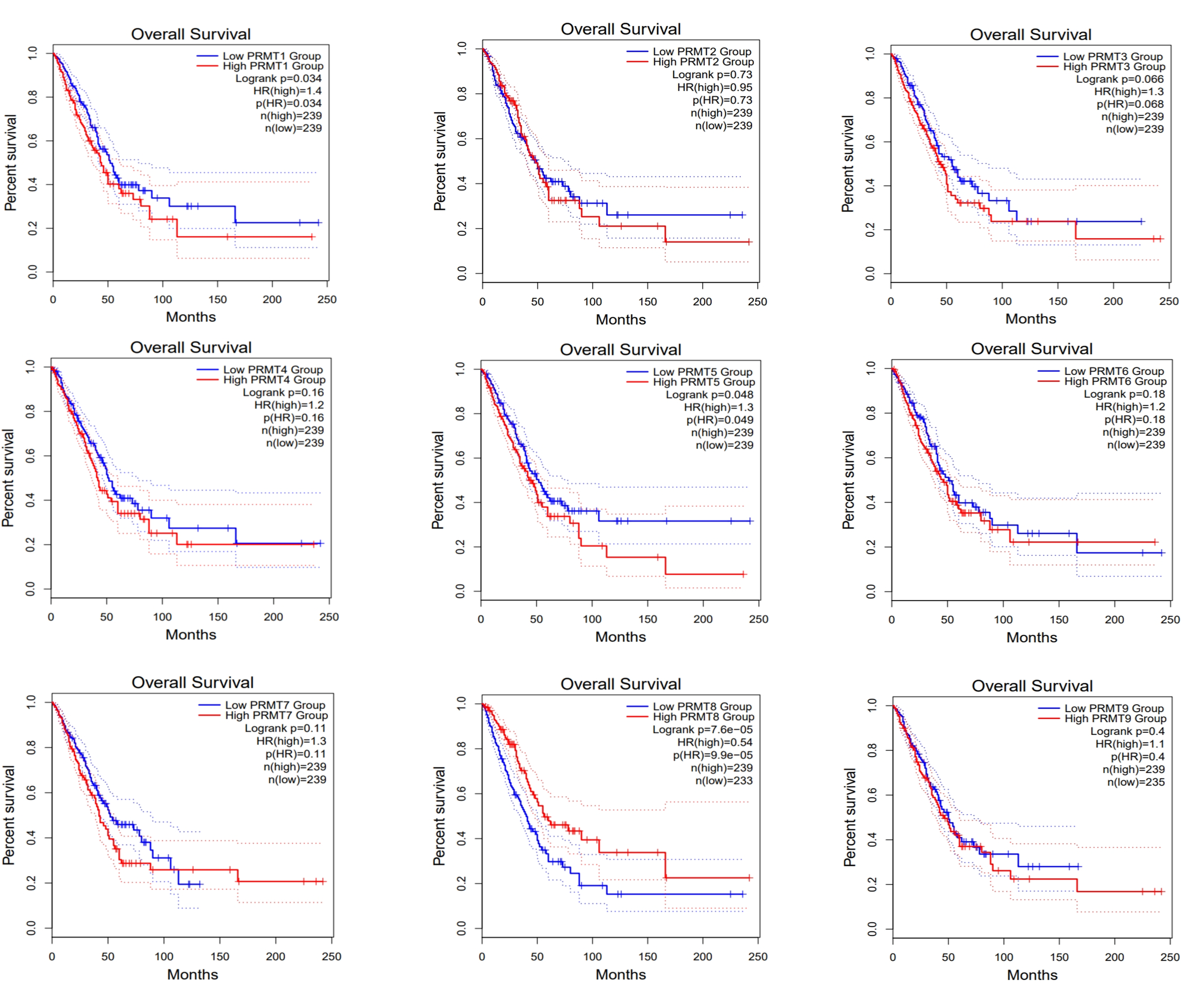

Supplement: Supplementary file 1 — Supplementary Material 1: Fig. S1. The prognostic analysis of PRMTS in LUAD patients by the GEPIA2 database. [file 12672_2025_2549_MOESM1_ESM.tif]

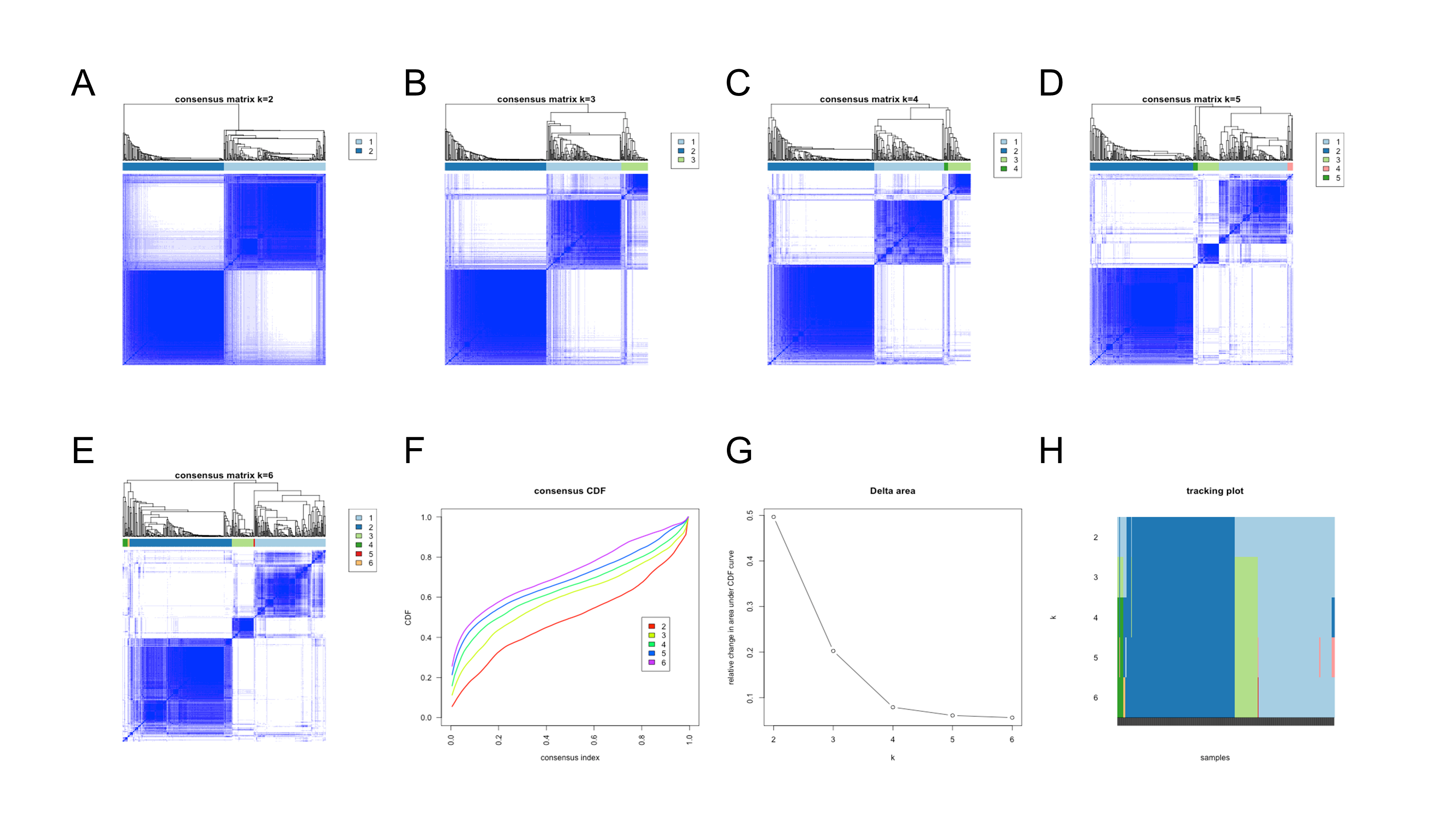

Supplement: Supplementary file 2 — Supplementary Material 2: Fig. S2. Identification of arginine methylation modification patterns based on the expression levels of the 9 PRMTs. (A-E) Consensus matrices of LUAD patients from k = 2 to k = 6. (F-H) The CDF curves plot, delta plot, and tracking plot corresponding to the consensus matrices from k = 2 to k = 6. [file 12672_2025_2549_MOESM2_ESM.tif]

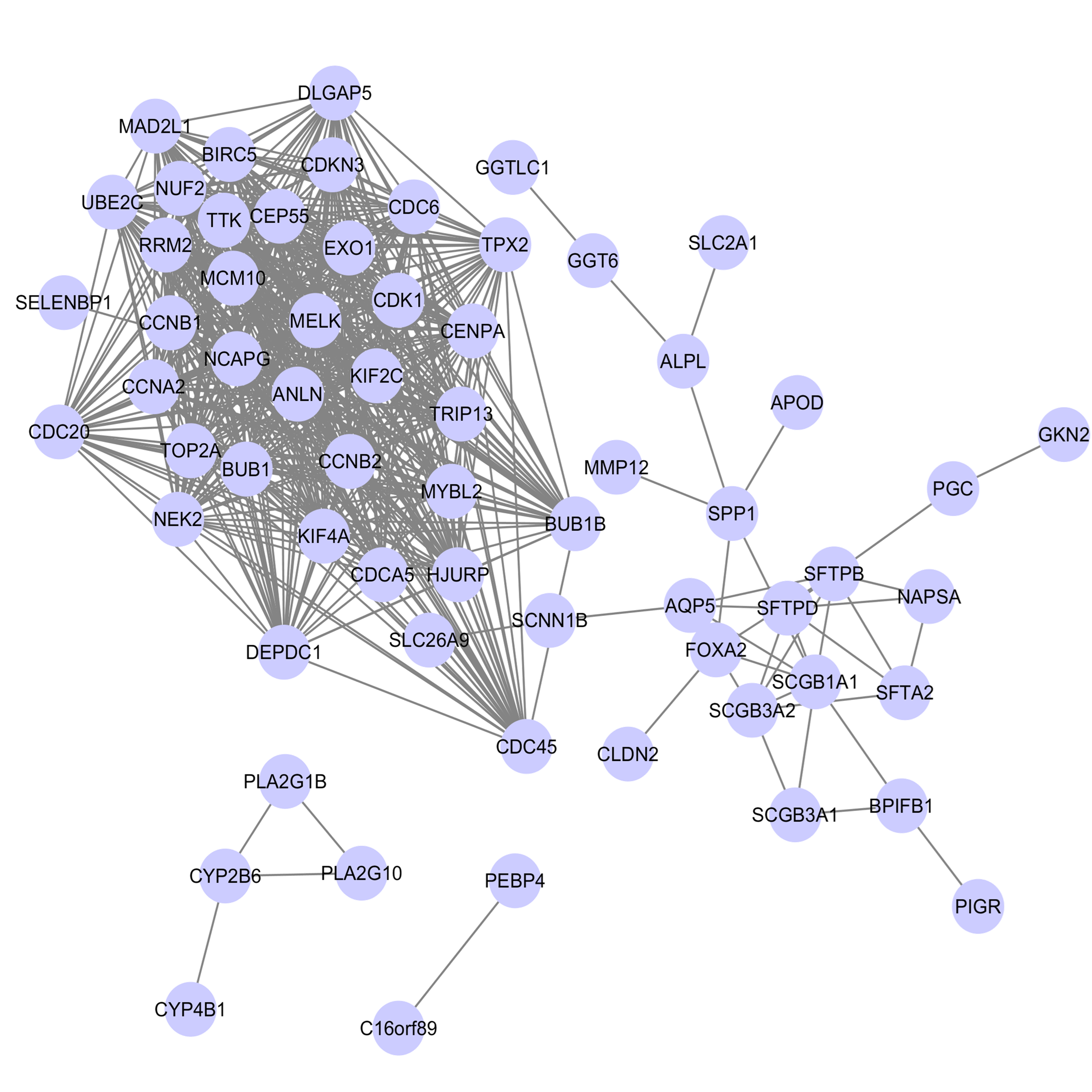

Supplement: Supplementary file 3 — Supplementary Material 3: Fig. S3. The PPI network of 79 DEG. [file 12672_2025_2549_MOESM3_ESM.tif]

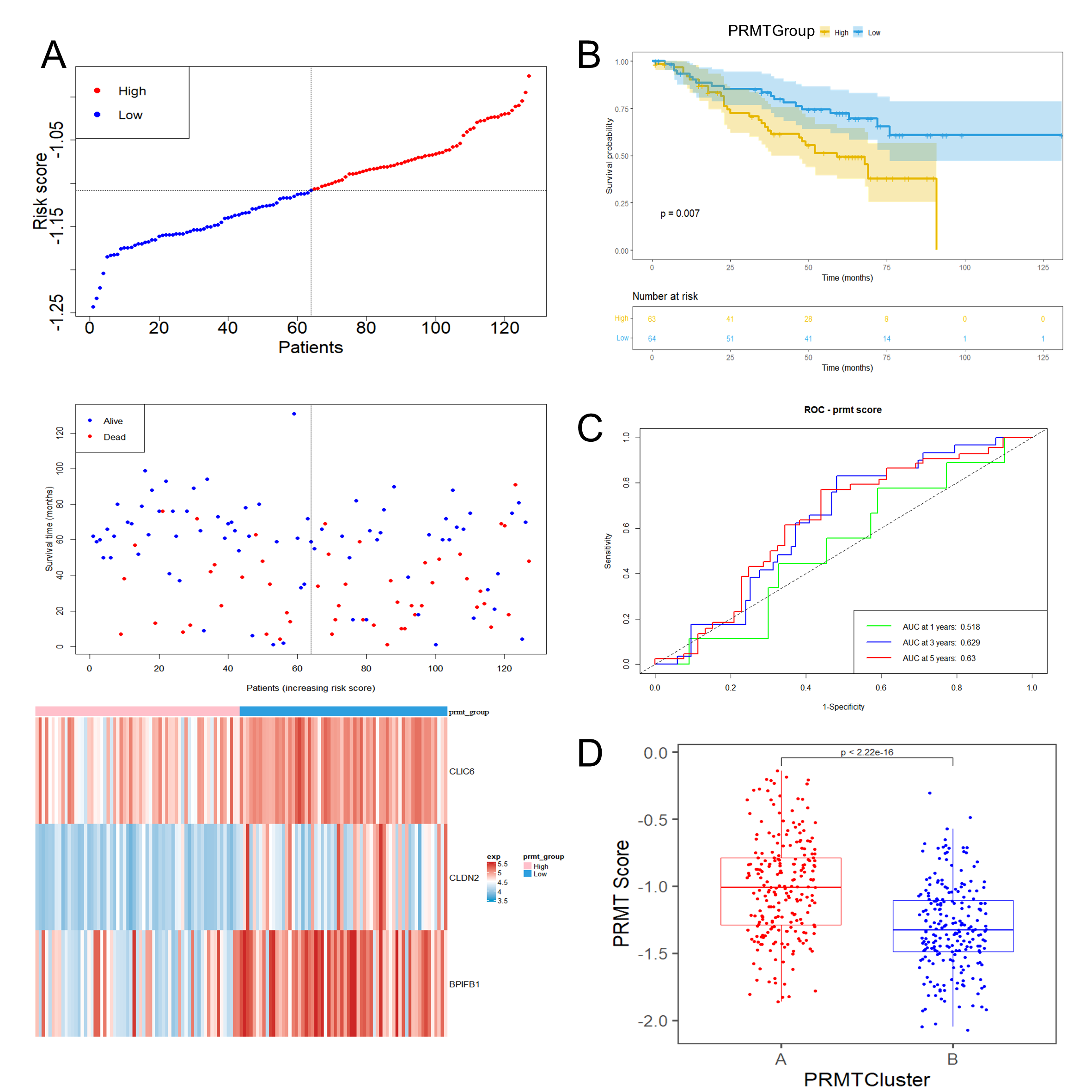

Supplement: Supplementary file 4 — Supplementary Material 4: Fig. S4. Verification of PRMTs-related prognostic model in the testing set. (A–C) The distributions, survival status, and expressions of the three prognostic genes in the testing set. (B) KM analysis for two PRMTGroups patients in the testing set. (C) ROC curves analysis in the in the testing set. (D) Differences of PRMTScore among two PRMTClusters. [file 12672_2025_2549_MOESM4_ESM.tif]

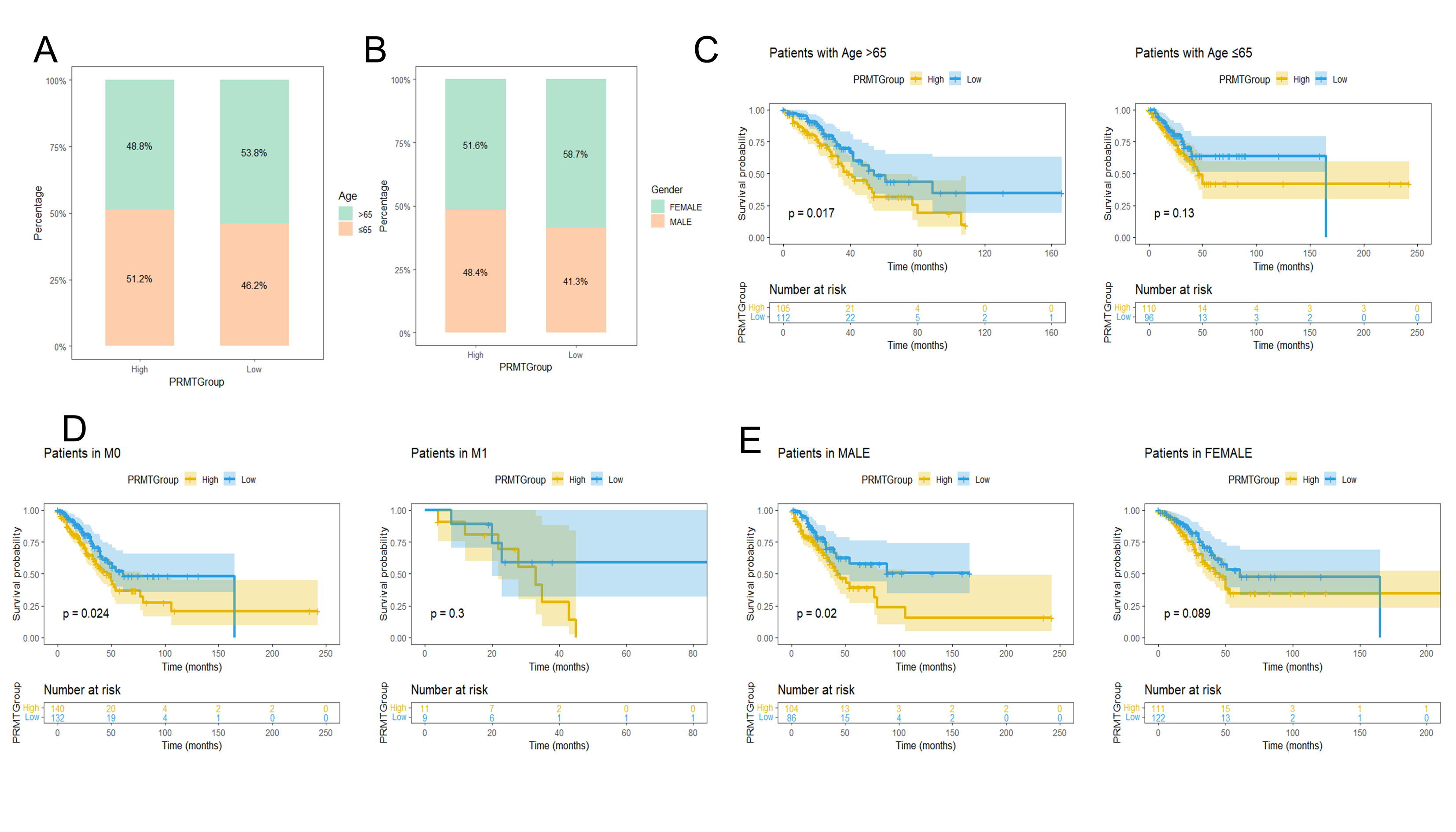

Supplement: Supplementary file 5 — Supplementary Material 5: Fig. S5. Correlation between clinical features and the prognosis. (A-B) The proportions of clinical features (age and gender) of two PRMTGroups patients. (C-E) KM analyses for two PRMTGroups patients with clinical features (age, M, and gender). [file 12672_2025_2549_MOESM5_ESM.tif]

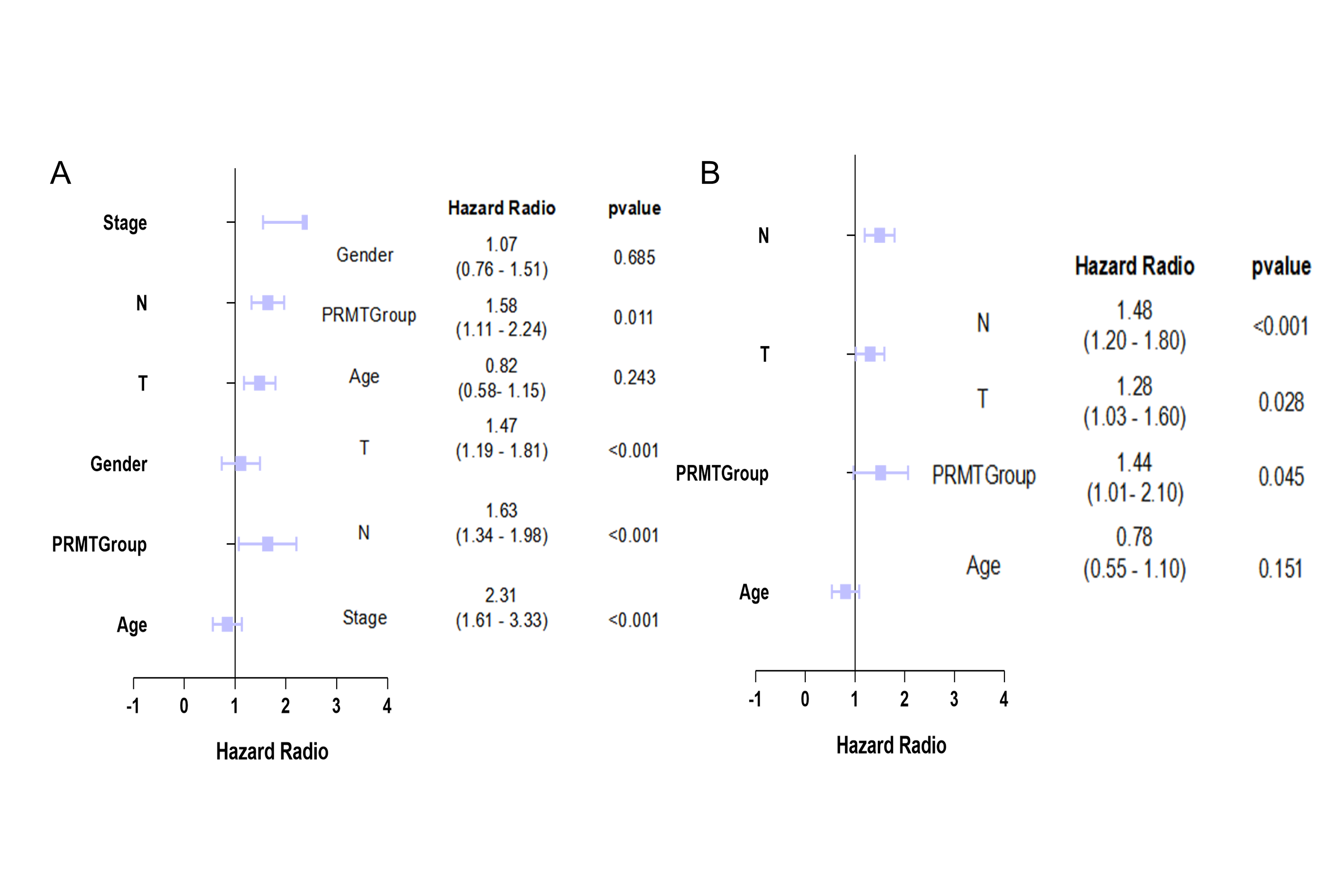

Supplement: Supplementary file 6 — Supplementary Material 6: Fig. S6. Univariate and multivariate Cox regression analyses based on the PRMT group and other clinical features in LUAD. (A) The univariate analysis. (B) The multivariate analysis. [file 12672_2025_2549_MOESM6_ESM.tif]

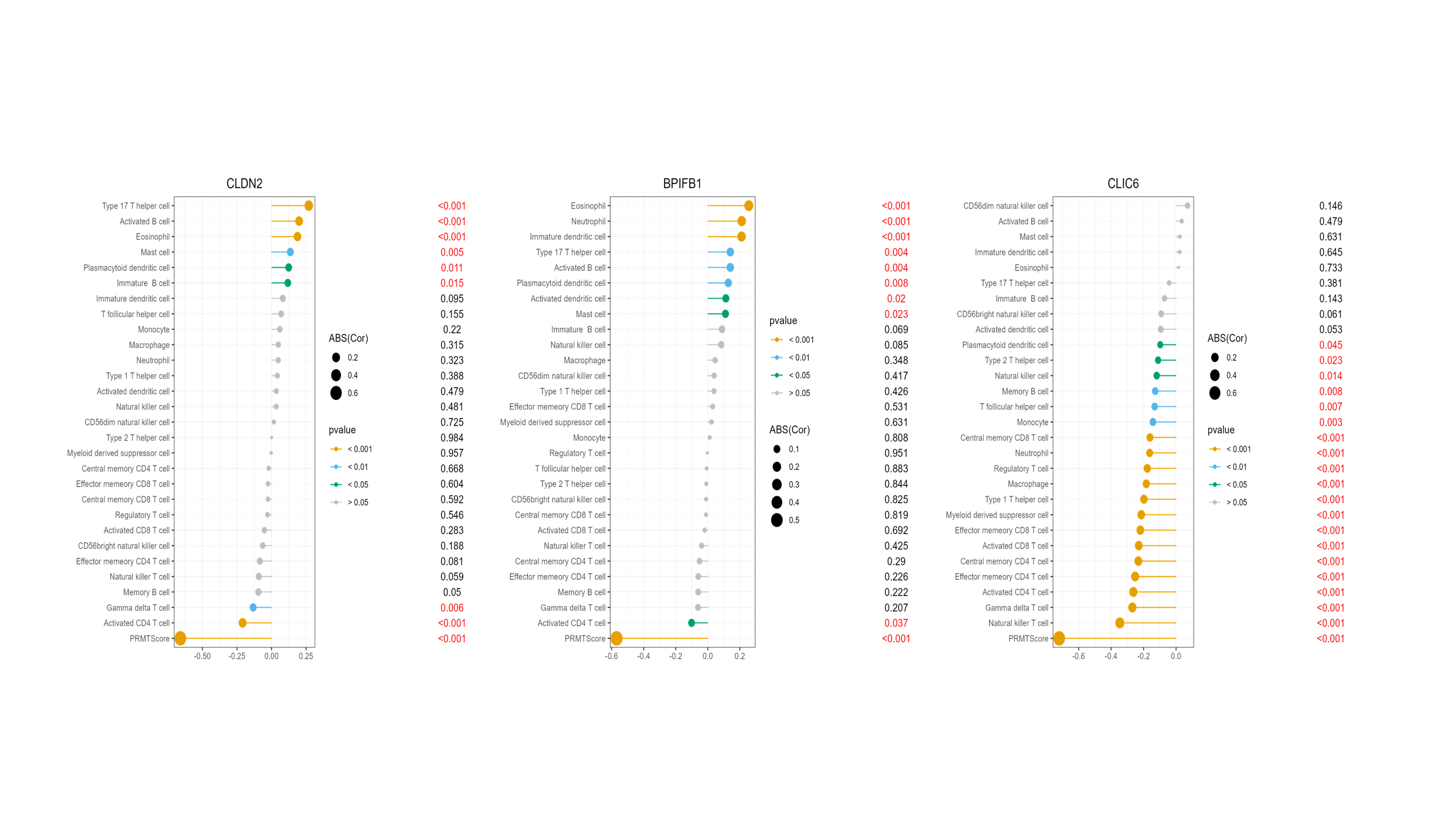

Supplement: Supplementary file 7 — Supplementary Material 7: Fig. S7. Correlation analysis of the PRMT-related genes (CLIC6, CLDN2, and BPIFB1) and immune cells. [file 12672_2025_2549_MOESM7_ESM.tif]
